# Blockade of mitochondrial components release by exosome pathway promotes the pathogenesis of Fuchs endothelial corneal dystrophy

**DOI:** 10.1038/s41420-025-02881-3

**Published:** 2025-12-02

**Authors:** Can Zhao, Qun Wang, Qingjun Zhou, Zhiqing Wang, Shuangqing Yao, Tian Sang, Haoyun Duan, Jingyi Wu, Xiaowei Zhong, Xin Sui, Weiyun Shi, Ting Wang

**Affiliations:** 1 Eye Institute of Shandong First Medical University, Qingdao, China; 2 Eye Hospital of Shandong First Medical University (Shandong Eye Hospital), Jinan, China; 3 State Key Laboratory Cultivation Base, Shandong Key Laboratory of Eye Diseases, Qingdao, China; 4 School of Ophthalmology, Shandong First Medical University, Jinan, China; 5 The First School of Clinical Medicine, Binzhou Medical University, Binzhou, China

**Keywords:** Corneal diseases, Experimental models of disease

## Abstract

Fuchs endothelial corneal dystrophy (FECD) is the leading indication of corneal transplantation worldwide and the focus of pathogenesis has been on the corneal endothelium. Instead of cellular analysis, we aimed to identify the protein changes of aqueous humor (AH) in patients with FECD and investigate in more detail the relationship between AH and corneal endothelium. We collected 13 AH samples of 7 early/middle stage FECD patients and 6 control patients during routine cataract surgery. The proteomes of AH were profiled with the 4D label-free quantitative tandem mass spectrometry. Among 1613 identified proteins, 44 proteins exhibited above two-fold upregulation in the AH of FECD patients than control patients. Gene ontology (GO) analysis showed the enrichment of mitochondrial components, which were further validated by ELISA of mitochondrial proteins SLC25A3, PC, and PARK7. Moreover, immunofluorescence staining and ultrastructural observation were conducted in clinical specimens, mouse corneal endothelium and cultured human corneal endothelial cells (HCECs). The mitochondrial protein TOM20 was reduced in the FECD corneal endothelium, accompanied by damaged mitochondrial ejection. We next isolated extracellular vesicles by ultracentrifugation from HCECs and revealed that the mitochondria copy numbers were significantly increased in UVA-irradiated cells. Inhibition of exosome biogenesis aggravated cell death and mitochondrial membrane potential impairment in FECD endothelial cells. Taken together, our results provided novel insights into the proteome characterization of the AH from FECD patients and offered new perspective to deepen the impaired mitochondrial quality control in the pathogenesis of FECD.

## Introduction

Fuchs endothelial corneal dystrophy (FECD) is the most prevalent primary corneal endothelial dystrophy and the predominant indication for corneal transplantation globally [1, 2]. It is characterized by the progressive decline of corneal endothelial cells (CECs), which leads to apoptosis, variation in size and shape of CEC morphology, decreased endothelial cell density, and the formation of extracellular matrix excrescences called guttae [3, 4]. Typically, FECD manifests in the fifth or sixth decade of life, with a higher incidence observed in women [5]. Its pathogenesis is hypothesized to result from an interplay between genetic predispositions and environmental factors [6]. Previous studies have linked FECD to an imbalance and dysregulation in the oxidative stress response [7]. Furthermore, oxidative stress and the subsequent accumulation of mitochondrial and nuclear DNA damage are pivotal in the pathogenesis of FECD, contributing significantly to CEC apoptosis and degeneration [8].

Mitochondria, often referred to as the powerhouses of cells, are responsible for generating adenosine triphosphate (ATP) through the electron transport chain and oxidative phosphorylation. The corneal endothelium harbors a high density of mitochondria to ensure the production of substantial ATP required for the Na^+^/K^+^-ATPase pump function [9]. In the FECD corneal endothelium, mitochondria exhibit a fission-dominant morphology and reduced mitochondrial density, attributed to the upregulation of mitophagy as part of the quality control mechanisms against defective mitochondria [10]. The fate of these damaged mitochondria has been elucidated in recent studies, suggesting that mitochondrial fragments can be packaged into extracellular vesicles (EVs) and expelled from the cell. This process has been observed in several physiological contexts and is induced in response to mitochondrial damage [11–14]. However, it remains unknown whether this process occurs in CECs or regulates FECD.

The aqueous humor (AH) provides essential support to the avascular tissues of the anterior segment of the eye, maintains intraocular pressure, and potentially modulates the pathogenesis of various ocular diseases [15–20]. Previous studies probed the AH proteome for alterations correlating with bullous keratopathy [21], penetrating keratoplasty-induced corneal endothelial decompensation and late-stage FECD [22]. Considering that FECD is a slowly evolving and progressive disease, a comprehensive analysis of AH from early/middle stage FECD is necessary.

In this study, we examined the alterations in the AH proteome associated with early/middle stage FECD. To identify significant enrichment in mitochondrial components, we conducted the enzyme-linked immunosorbent assay (ELISA) on AH clinical specimens, informed by bioinformatic analysis. Furthermore, we employed transmission electron microscopy (TEM) and mitochondrial labeling techniques in an FECD cellular model to visualize the release of mitochondria from CECs. More interestingly, we have preliminary confirmed that CECs released damaged components of mitochondria via extracellular vesicles. This work would provide theoretical insights and clinical implications for the treatment of FECD.

## Results

### Demographic data

**Fig. 1:**
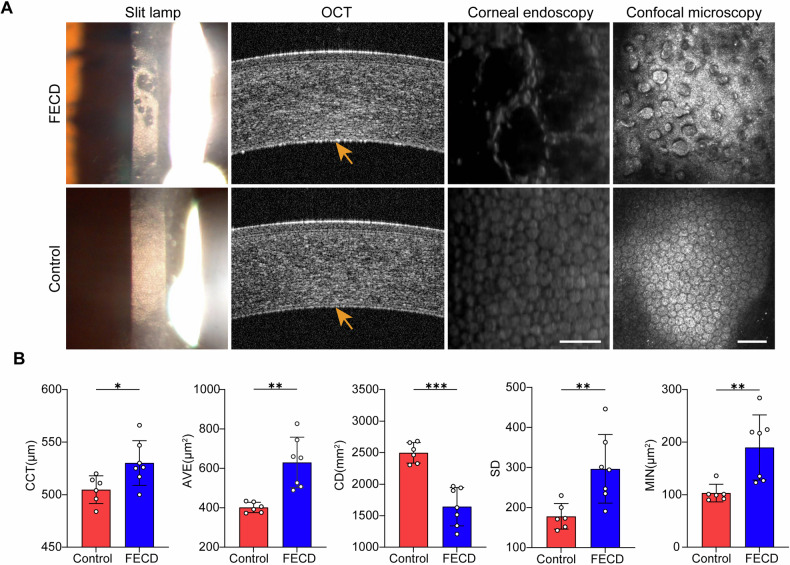
Comparisons of corneal endothelial morphology in FECD and control patients. **A** Representative images of slit lamp, optical coherence tomography (OCT), corneal endoscopy, and confocal microscopy for FECD and control groups. Yellow arrows on the OCT images highlight dense and irregular hyper-reflective projections in FECD corneal endothelium. Scale bar, 50 μm. **B** Quantitative analysis of central corneal thickness (CCT), average cell area (AVE), cell density (CD), cell area standard deviation (SD), and minimum cell area (MIN) in FECD and control groups (n = 6 for control, *n* = 7 for FECD. CCT: *P* = 0.028, AVE: *P* = 0.001, CD: *P* < 0.001; SD: *P* = 0.008; MIN: *P* = 0.007). Data are shown as mean ± SD. The significance of differences was detected using unpaired two-sided Student’s *t* test, *P < 0.05, **P < 0.01, ***P < 0.001.

This study included 7 patients diagnosed with FECD (3 males and 4 females) with a mean age of 64.86 ± 4.3 years. The control group consisted of 6 age-matched senile cataract patients (3 males and 3 females) with an average age of 64.33 ± 3.93 years (Table 1). First, slit-lamp microscopy demonstrated the endothelial abnormalities in FECD patients. Furthermore, corneal optical coherence tomography (OCT) examination revealed the central corneal thickening and a high density of guttae on the Descemet’s membrane (DM) surface in FECD patients, whereas the DM of the control group appeared flat and uniform. Specular microscopy of the corneal endothelium in FECD patients showed “black areas”, indicating endothelial cell loss. Confocal microscopy further disclosed the presence of dense guttae on the DM surface of FECD patients, with CECs exhibiting deformation or being absent altogether (Fig. 1A).

**Table 1:** Demographic and clinical status of patients.

Characteristics	FECD	Normal	*P*
Number	7	6	--
Age	64.86 ± 4.3	64.33 ± 3.93	0.396
Gender (M/F)	3/4	3/3	0.452
LogMAR BCVA	0.41 ± 0.14	0.38 ± 0.13	0.105
IOP (mmHg)	12.86 ± 2.97	14.33 ± 2.66	0.368
AL (mm)	22.84 ± 0.78	22.92 ± 0.86	0.864
CCT (μm)	530.14 ± 21.19	504.83 ± 13.15	0.028
CD (cells/mm^2^)	1643.00 ± 303.94	2497.50 ± 161.86	<0.001
6 A (%)	46.71 ± 12.37	46.00 ± 5.02	0.816
CV	44.71 ± 5.71	46.83 ± 4.71	0.968
AVE (μm^2^)	630.00 ± 128.38	401.83 ± 26.23	0.001
MAX (μm^2^)	1285.57 ± 320.66	1161.33 ± 248.70	0.457
MIN (μm^2^)	189.86 ± 61.97	103.17 ± 16.75	0.007
SD (μm^2^)	296.71 ± 85.79	178.17 ± 32.13	0.008

Data are showed with mean ± SD.

*BCVA* best corrected visual acuity, *IOP* intraocular pressure, *AL* axial length, *CCT* central corneal thickness, *CD* cell density, *6* *A* percentage of hexagonal cells, *CV* cell area coefficient of variation, A*VE* average cell area, *MAX* maximum cell area, *MIN* minimum cell area, *SD* cell area standard deviation.

Quantitative analysis revealed that the corneal endothelial cell density (CD) in the FECD group was 1643.00 ± 303.94 cells/mm^2^, significantly lower than that in the control group (*P* < 0.001). The central corneal thickness (CCT) in FECD patients was 530.14 ± 21.19 μm, compared to 504.83 ± 13.15 μm in the control group, displaying a statistically significant difference (*P* = 0.028). Specular microscopy revealed marked variations in average cell area (AVE) and minimum cell area (MIN) between the FECD and control groups (*P* = 0.001; *P* = 0.007). However, no differences were observed in best corrected visual acuity (BCVA), axial length (AL), percentage of hexagonal cells (6 A), cell area coefficient of variation (CV) and maximum cell area (MAX) between the two groups (*P* > 0.05) (Table 1 and Fig. 1B).

### Proteomic profiling and functional enrichment analysis of aqueous humor from FECD and control patients

**Fig. 2:**
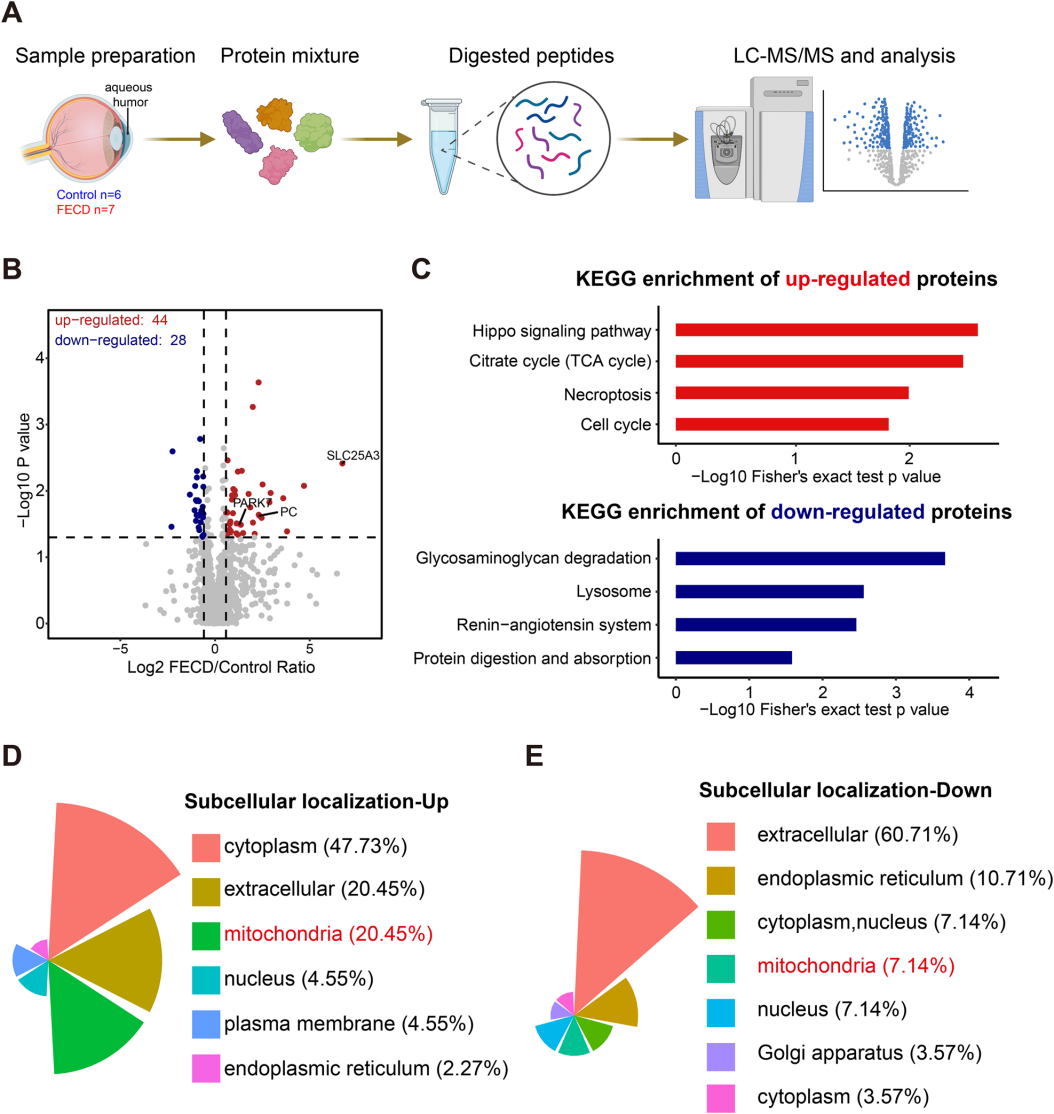
Proteomic profiling of the aqueous humor in FECD patients. **A** Overview of the study workflow. Control, *n* = 6; FECD, *n* = 7. **B** Volcano plots showing DEPs in AH of control and FECD patients. The threshold set as fold change > 2, *P* < 0.05. **C** The enriched KEGG pathways in upregulated proteins and downregulated proteins. **D**, **E** The polar area diagram showing subcellular localization of DEPs in AH of control and FECD patients.

The label-free LC-MS/MS and proteomic analysis was performed as the following workflows (Fig. 2A). After filtering out weakly expressed proteins and multiple analyses, a total of 72 differentially-expressed proteins (DEPs) were identified. In the FECD group, 44 proteins exhibited more than twofold upregulation, and 28 proteins showed at least twofold downregulation compared to the control group (Fig. 2B). To further characterize the pathological changes of FECD AH, KEGG analysis was performed. As shown in Fig. 2C, the enriched pathway of the upregulated proteins included Hippo signaling pathway, the tricarboxylic acid (TCA) cycle, necroptosis, and cell cycle, while the down-regulated proteins mainly associated with lysosome and protein digestion and absorption. These activated pathways suggested potential metabolic dysregulation in FECD CECs, and the lysosomal downregulation likely contributed to compromised autophagy under FECD conditions. It must be noted that the proteomic changes in the AH do not fully reflect the pathological alterations occurring in corneal endothelial cells. This may be attributed to the complex origins of the aqueous humor. Moreover, our aqueous humor samples were obtained from patients with early to mid-stage FECD, rather than late-stage disease, in which aberrant activation of autophagy is more pronounced and may not be clearly detectable in the earlier stages.

Then, using the subcellular localization prediction, we found that among the upregulation proteins, 47.73% were predicted as cytoplasmic proteins, 20.45% were extracellular proteins, and 20.45% were annotated as mitochondrial proteins. However, downregulated proteins were primarily extracellular (60.71%) with only 7.14% mitochondrial representation. These changes in protein distribution, including reduced extracellular proteins coupled with increased intracellular proteins, reflect a combined effect that may disrupt cellular homeostasis and contribute to the progression of FECD. Moreover, the overall trend is a significant enrichment of mitochondrial components in the AH of FECD patients. Given that FECD is a disorder of mitochondrial dynamics, we sought to further confirm and investigate the potential regulatory role of mitochondrial components in AH during FECD pathogenesis.

### The identification of upregulated mitochondrial components in AH during FECD pathogenesis

**Fig. 3:**
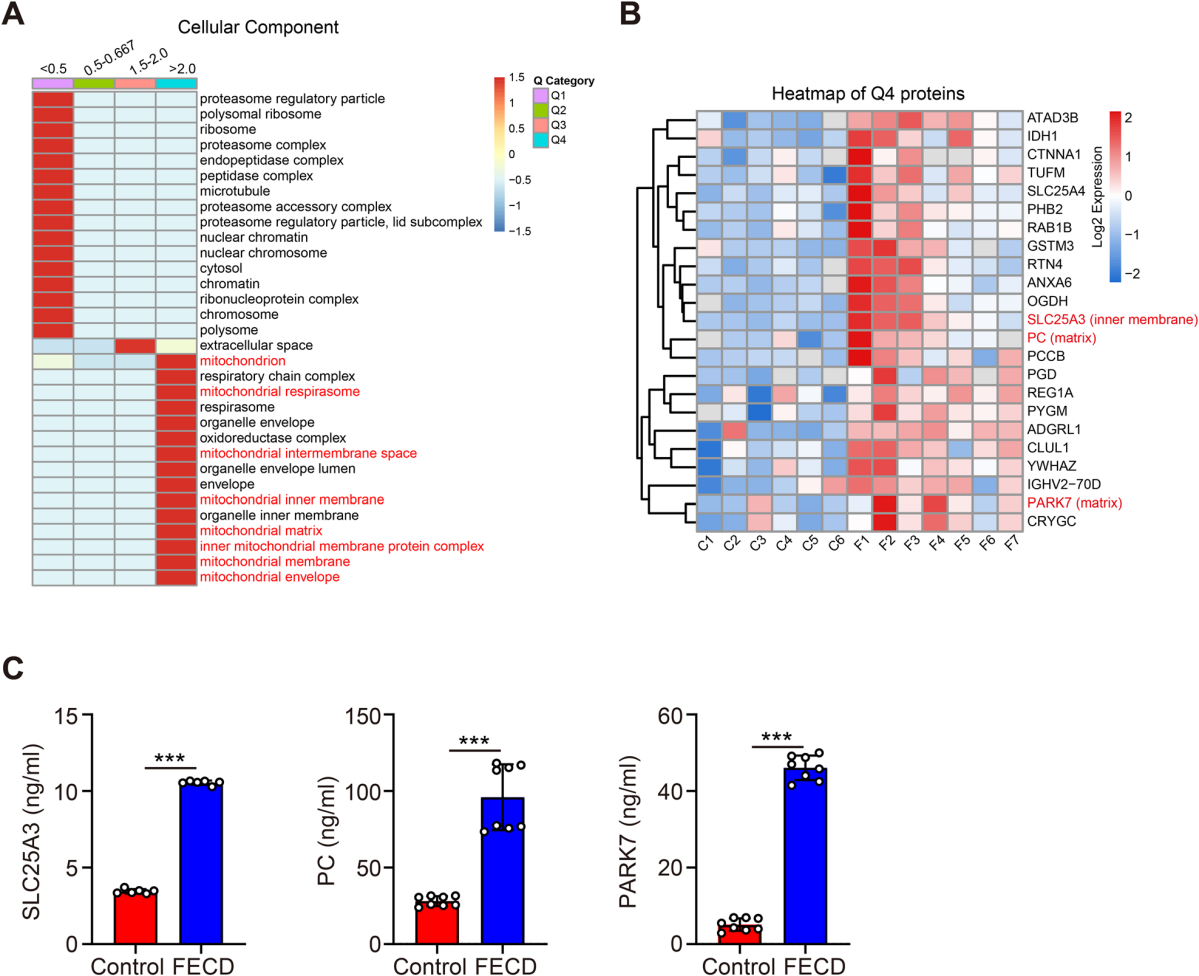
Mitochondrial proteins analysis of the aqueous humor from patients with and without FECD. **A** Four sets of hierarchical clustering analysis for enrichment of DEPs cellular component. **B** Heatmap depicts the DEPs of Q4 cluster. C = control; F = FECD. **C** ELISA verified the upregulation of proteins associated with mitochondrial function in FECD AH (SLC25A3: *n* = 6 samples per group, *P* < 0.001; PC: *n* = 8 samples per group, *P* < 0.001; PARK7: *n* = 8 samples per group, *P* < 0.001). Data are shown as mean ± SD. The significance of differences was detected using unpaired two-sided Student’s *t*-test, ***P < 0.001.

For further hierarchical clustering based on DEPs functional classification, we divided them into 4 categories according to their differential expression folds, called Q1 to Q4 ( <0.5, 0.5–0.667, 1.5–2.0, >2.0, respectively). Then the cellular component was enriched separately for each group, and cluster analysis was performed to find the correlation of protein functions with differential expression folds in the comparison groups. As shown in Fig. 3A, data suggested that DEPs in AH linked to the mitochondrial components and mitochondrial function were highlighted in the Q4 cluster. A heatmap of proteins from Q4 cluster is shown in Fig. 3B and to confirm the reliability of the identified proteins in the FECD AH, 3 candidates including solute carrier family 25 member 3 (SLC25A3, mainly located in mitochondrial inner membrane), pyruvate carboxylase (PC, mainly located in mitochondrial matrix), and parkinsonism associated deglycase (PARK7, mainly located in mitochondrial matrix) were selected and evaluated using ELISA analysis. Emerging evidence reported that SLC25A3 [23, 24], PC [25, 26], and PARK7 [27, 28] contributed to preserving mitochondrial function and metabolic homeostasis. Consistent with the proteomic data, we detected significantly upregulated SLC25A3, PC, and PARK7 in FECD AH (Fig. 3C). In this regard, our results supported the reliability of upregulated mitochondrial components in AH during FECD pathogenesis.

### More release of mitochondrial proteins outside the cell during FECD pathogenesis

**Fig. 4:**
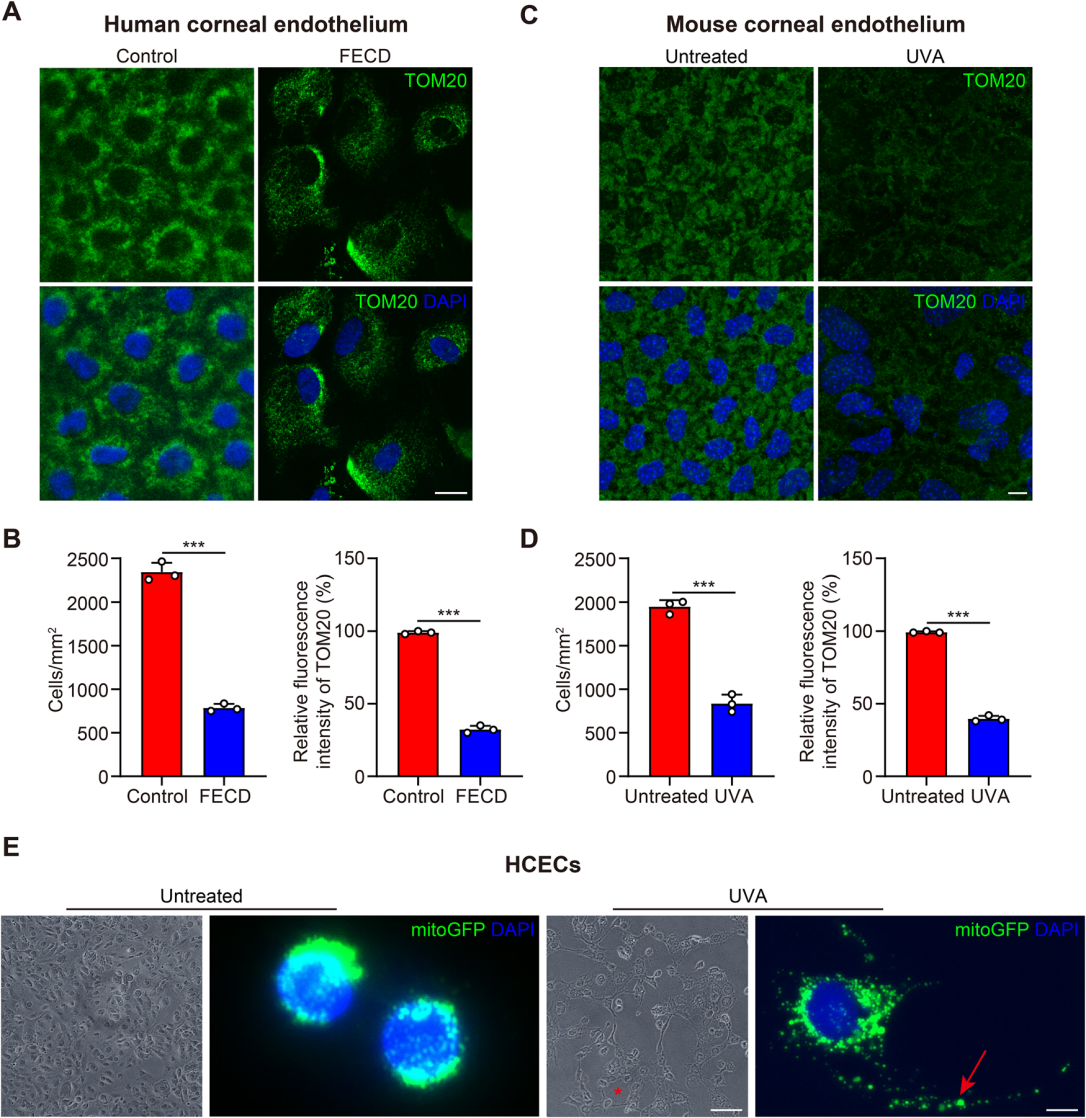
Enhanced release of extracellular mitochondria in FECD CECs. **A** Representative confocal images of whole mount of corneal endothelium detecting TOM20 in CECs of FECD and normal samples. Scale bar, 10 μm. The experiment was repeated at least three times independently with a similar result. **B** TOM20 immunostaining-based analysis for cell density and the relative fluorescence intensity in human corneal endothelium (*n* = 3 samples per group. cell density: *P* < 0.001; relative fluorescence intensity: *P* < 0.001). **C** TOM20 staining showed the mitochondria impairment in corneal endothelium of in normal and FECD mouse. Scale bar, 10 μm. The experiment was repeated at least three times independently with a similar result. **D** Analysis for cell density and the relative fluorescence intensity in mouse corneal endothelium (*n* = 3 samples per group. cell density: *P* < 0.001; relative fluorescence intensity: *P* < 0.001). **E** Representative images of HCECs expressing MitoGFP treated with 50 J/cm^2^ UVA irradiation. Scale bar: left 100 μm, right 10 μm. Data are shown as mean ± SD. The significance of differences was detected using unpaired two-sided Student’s *t*-test, ***P < 0.001.

To investigate the potential role of mitochondrial components in AH during FECD pathogenesis, we first attempted to evaluate the mitochondrial impairment in CECs. TOM20 is a hallmark of the mitochondrial outer membrane. It is noteworthy that TOM20 expression was robustly positive and densely distributed around the nucleus in healthy corneal endothelium (Fig. 4A). Conversely, in FECD patients, the CECs density was markedly reduced. Moreover, TOM20 expression was significantly diminished and appeared sparsely and loosely distributed within the cytoplasm (Fig. 4B). We then established a non-genetic FECD mouse model whose corneal exposure to UVA recapitulated the symptoms of FECD. Previous studies have shown that corneal exposure to UVA effectively mimics the morphological and molecular features of FECD, including mitochondrial dysfunction, loss of endothelial cells, and the formation of guttae, which are characteristics of FECD pathology [29–31]. Consistent with clinical samples, the CEC density in the UVA-irradiated mice was reduced, and TOM20 expression in the corneal endothelial cells was significantly decreased (Fig. 4C, D). To trace mitochondrial dynamics in FECD, we transfected HCECs with the Mito-GFP lentivirus [32] and subjected them to UVA irradiation. In the control group, Mito-GFP was densely localized within the cytoplasm, predominantly around the nucleus, whereas in the FECD group, the translocation of mitochondria was observed, and they tended to move outside the HCECs (Fig. 4E). These data prompted us to further detect the spatial localization and release form of mitochondria during FECD pathogenesis.

### Enhanced release of extracellular mitochondria in FECD endothelial cells

**Fig. 5:**
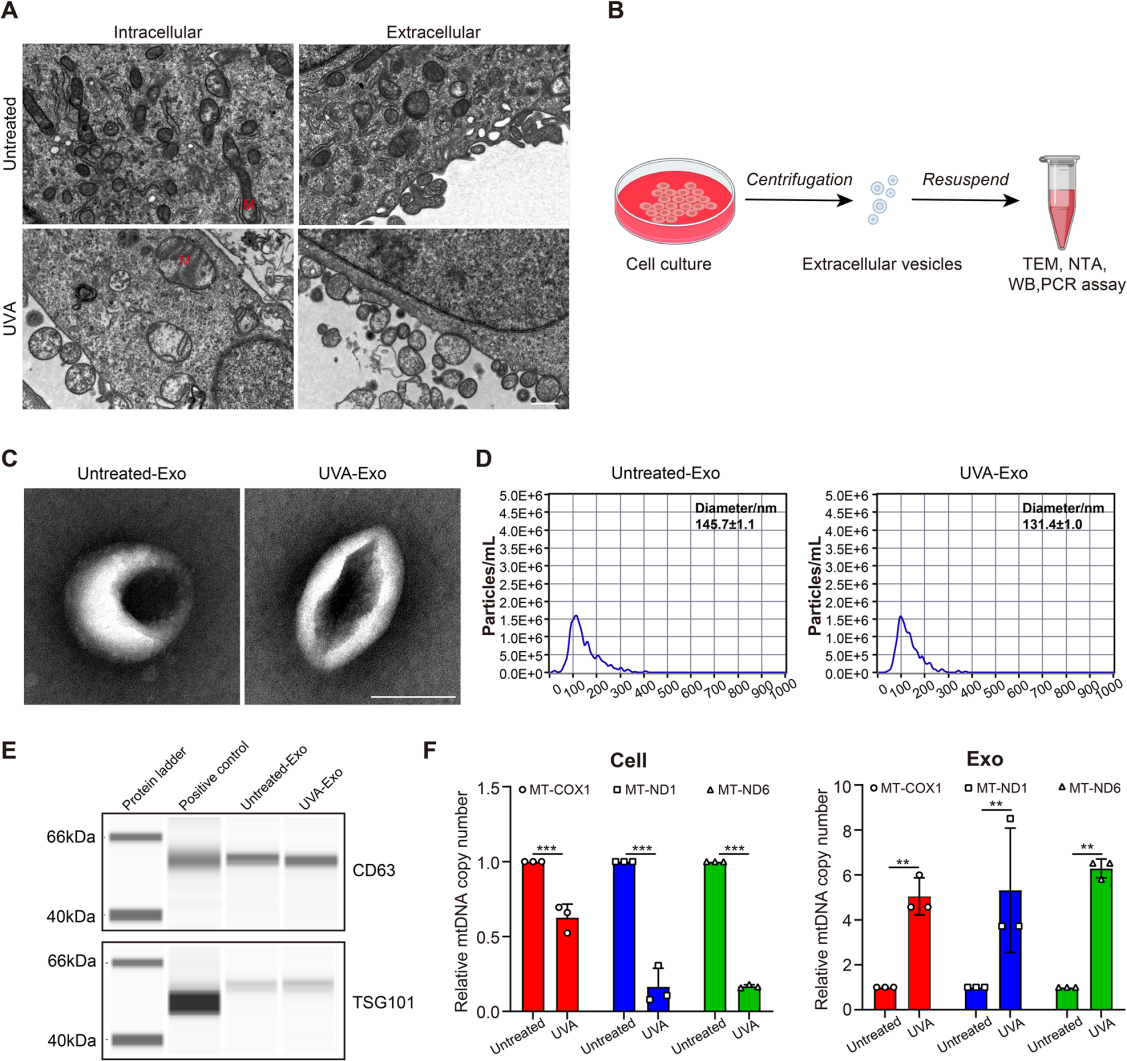
Mitochondrial positioning and release type in HCECs with or without UVA exposure. **A** Representative TEM images of HCECs treated with UVA irradiation. Scale bar, 2 μm. **B** The schematic procedure of isolation and identification of extracellular vesicles. **C** Representative TEM images of exosomes from HCECs in the presence or absence of UVA irradiation. Scale bar, 100 nm. **D** Size distributions of exosomes derived from HCECs in the presence or absence of UVA determined using NAT. **E** WB analysis of EV markers in different groups. **F** Mitochondrial abundance by qPCR in HCECs and isolated exosomes upon UVA treatment (Cell: *n* = 3 samples per group, MT-COX1, *P* < 0.001, MT-ND1, *P* < 0.001, MT*-*ND6, *P* < 0.001; Exo: *n* = 3 samples per group, MT-COX1, *P* = 0.004, MT-ND1, *P* = 0.0024, MT*-*ND6, *P* = 0.004). Exo exosome. Data are shown as mean ± SD. The significance of differences was detected using unpaired two-sided Student’s *t*-test, **P < 0.01, ***P < 0.001.

Firstly, TEM analysis revealed a dramatic accumulation of extracellular vehicles (EVs), implying the release form of mitochondria and the increased EVs secretion with UVA exposure (Fig. 5A). To verify this observation biochemically, the EVs were isolated by ultracentrifugation, using standard procedures (Fig. 5B). As shown in Fig. 5C, the TEM images revealed that the exosomes obtained from HCECs in the presence or absence of UVA irradiation displayed a representative exosome morphology with a round or cup-shaped structure. The size of exosomes from different origins was evaluated using nanoparticle tracking analysis (NTA) (Fig. 5D). Those isolated from HCECs with or without UVA treatment showed a bell-shaped curve, with an average exosome size of 145.7 ± 1.1 nm (Untreated-Exo) and 131.4 ± 1.0 nm (UVA-Exo). Moreover, the significant expression of surface markers (CD63 and TSG101) in exosomes isolated from UVA-treated HCECs was observed through Western blot (WB), similar to exosomes obtained from untreated HCECs (Fig. 5E). Next, we detected the mitochondrial DNA (mtDNA) copy numbers of HCECs and exosomes. Eventually, we observed that mtDNA copy number is dramatically reduced in HCECs after UVA treatment. Conversely, mtDNA copy number does indeed increase in UVA-Exo, implying an enrichment of mitochondrial components (Fig. 5F). These data suggested that CECs can release more components of mitochondria via extracellular vesicles under mitochondrial stresses caused by FECD conditions.

### Aggravated cell death and reduced mitochondrial membrane potential in FECD endothelial cells with inhibition of exosome biogenesis

**Fig. 6:**
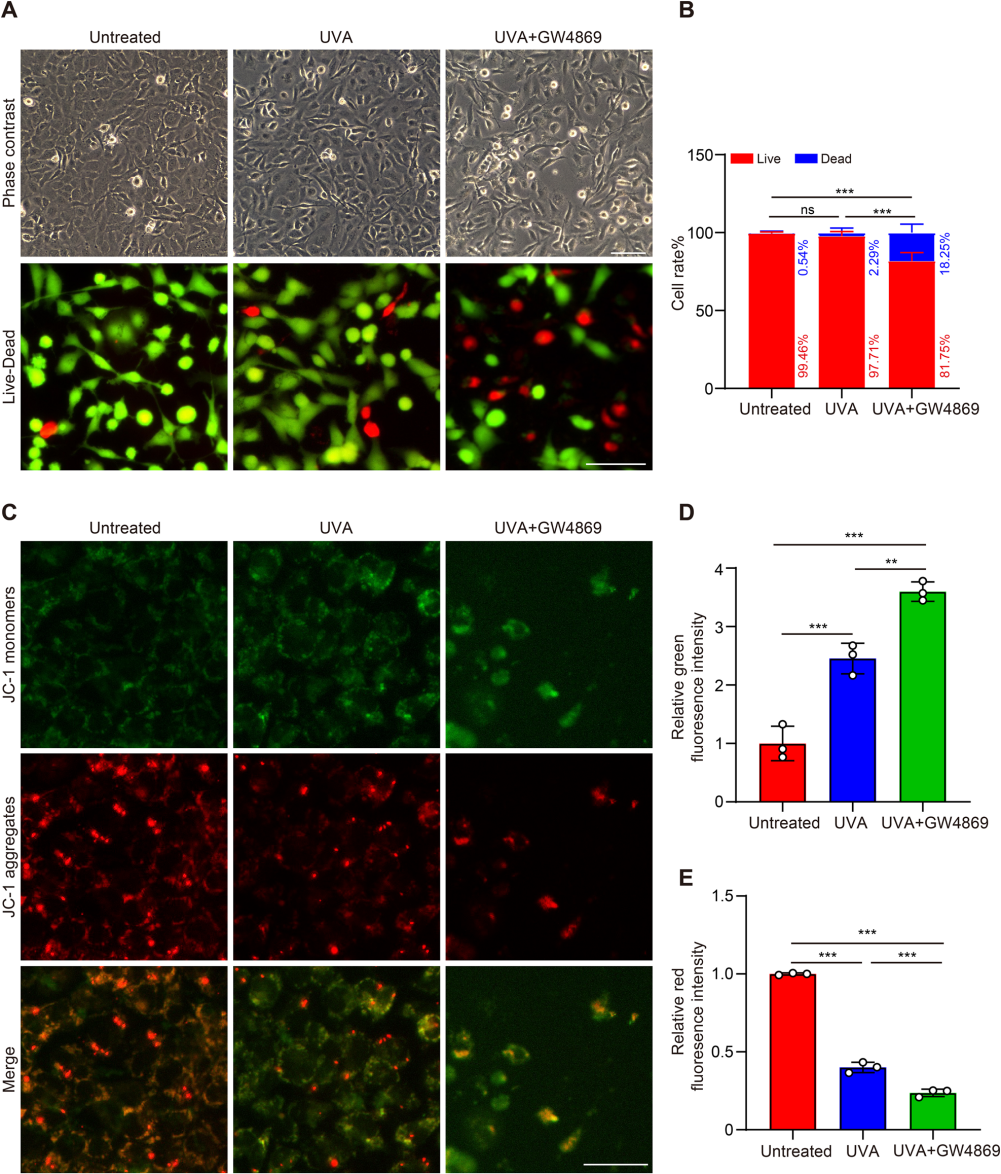
Cell viability and mitochondrial function of FECD HCECs with or without GW4869. **A** Representative bright field and immunofluorescence images of Live-Dead staining. Green: Living cells; Red: Dead cells. Scale bar, 90 μm (top) and 65 μm (below). **B** Percentage of living and dead cells in FECD HCECs with or without GW4869 (*n* = 4 samples per group. Untreated vs UVA *P* = 0.8776, Untreated vs UVA + GW4869 *P* < 0.001, UVA vs UVA + GW4869 *P* < 0.001). **C** Representative immunofluorescence staining of JC-1. Green: monomeric form; Red: aggregated form. Scale bar, 65 μm. Effects of UVA and GW4869 on relative JC-1 monomer (**D**) /aggregate (**E**) fluorescence intensity (*n* = 3 samples per group. Green: Untreated vs UVA *P* < 0.001, Untreated vs UVA + GW4869 *P* < 0.001, UVA vs UVA + GW4869 *P* = 0.0031; Red: Untreated vs UVA *P* < 0.001, Untreated vs UVA + GW4869 *P* < 0.001, UVA vs UVA + GW4869 *P* < 0.001). Data are shown as mean ± SD. The significance of differences was detected using one-way ANOVA followed by Dunnett’s test, **P < 0.01, ***P < 0.001, ns, not significant.

GW4869 is a potent inhibitor of neutral sphingomyelinase (nSMase), which blocks exosome biogenesis by preventing ceramide-dependent budding of intraluminal vesicles into multivesicular bodies [33, 34]. To validate the exosome-release pathway, we inhibited exosome biogenesis with GW4869, and cell viability and mitochondrial membrane potential were observed in FECD endothelial cells. As shown in Fig. 6A, HECEs treated with UVA irradiation exhibited a small amount of cell loss, while 20 μM GW4869 lead to the number of HCECs decreased perceptibly, and the cells became shriveled and rounded. Similarly, the Live-Dead assay demonstrated that UVA irradiation exhibited no change in the percentage of dead cells (Untreated versus UVA: 0.54 ± 0.99 versus 2.29 ± 2.94, *P* > 0.05), while GW4869 treatment increased this percentage to 18.25 ± 5.44 (**P < 0.01) (Fig. 6B). Δψm is a crucial indicator of mitochondrial health and function. Then, we conducted the JC-1 assay to assess Δψm in FECD HCECs. As shown in Fig. 6C–E, a red fluorescence was predominant in untreated cells, indicating the presence of JC-1 in the aggregated form in mitochondrial membranes. However, UVA-treated cells showed increased green fluorescence, indicating the existence of free JC-1 and the depolarized mitochondrial membrane potential. UVA plus GW4869-treated cells exhibited the weakest red fluorescence and robust green fluorescence. Collectively, our results suggested that blocking exosome biogenesis exacerbated cell death and mitochondrial dysfunction in FECD HCECs.

## Discussion

FECD represents a prevalent cause of corneal endothelial decompensation, leading to progressive vision impairment necessitating eventual corneal transplantation, which imposes substantial economic and social burdens. Therefore, identifying non-invasive therapeutic targets that can be addressed early to slow or halt disease progression holds significant promise for benefiting a large patient population. In this study, we employed 4D label-free quantitative proteomic analysis to investigate proteomic alterations in the AH of patients at the early/mid-stage of FECD. This exploration aims to elucidate the underlying mechanisms driving the onset and progression of FECD, thereby providing novel insights and strategies for the clinical prevention and management of disorders associated with the corneal endothelium.

Previous studies have highlighted the role of AH composition in the pathogenesis of FECD, albeit with limited depth and a focus primarily on end-stage disease. To advance early prevention and treatment strategies for FECD, for the first time, our study extended to investigate the correlation between AH proteomics and the pathogenesis of FECD during its early and middle stages. Unlike previous proteomics of late-stage FECD AH, the abundance of mitochondria-associated proteins was identified. It is well established that CECs exhibit altered mitochondria characterized by mitochondrial DNA damage, diminished mitochondrial membrane potential, attributable to constitutive activation of mitophagy in FECD [35]. We reasoned that if CECs release small amounts of damaged mitochondria under physiological conditions, while FECD caused more mitochondrial release to serve as a mitochondrial quality control mechanism for dealing with excessive oxidative stress. Indeed, we confirmed that more components of mitochondria were released from UVA-irradiated CECs by extracellular vesicles. At this point, further studies should dissect the release mechanism in detail.

It is well known that extracellular vesicles can function as signaling structure by delivering nucleotides or proteins to surrounding cells [36]. According to the literature, microvesicles enriched in mitochondria released by monocytic cells induced type I Interferons (IFN) and Tumor necrosis factor (TNF) signaling in endothelial cells after uptake [37]. Mitochondria phagocytosed by macrophages undergo fusion with the existing mitochondrial network and enhance respiration [38]. Moreover, secretion of extracellular vesicles in response to lysosomal impairment can function as an alternative quality control mechanism to dispose of cargo [39]. Hence, it could not be excluded that the increased mitochondrial components released by CECs in turn could contribute to the progression of FECD.

Evidence from studies on nerve cells, cardiomyocytes, mesenchymal stem cells and adipocytes has demonstrated the ability to expel damaged mitochondria from the cytosol [40–42]. Different cells possess different release forms or multiple forms. Neurons and cardiomyocytes can extrude protein aggregates and mitochondria in large vesicles (about 3.5–4 μm) [40, 43]. Mesenchymal stem cells can transfer mitochondria to macrophages in microvesicles secretion form [38]. Rosina et al. reported that brown adipocytes eliminate damaged mitochondrial parts through extracellular vesicles [42]. Given that CECs are post-mitotic and require long-term survival, stringent control over mitochondrial quality is imperative. The following study should also be extended to other types of mitochondrial release.

Due to the relatively low incidence of Fuchs’ corneal endothelial dystrophy in China, the sample size in this study is limited by the availability of eligible patients. Nevertheless, the conclusions derived from our sequencing data, supported by subsequent experimental validation, demonstrate the reliability of our findings. We believe that future studies with an expanded sample size could further substantiate and refine these results.

**Fig. 7:**
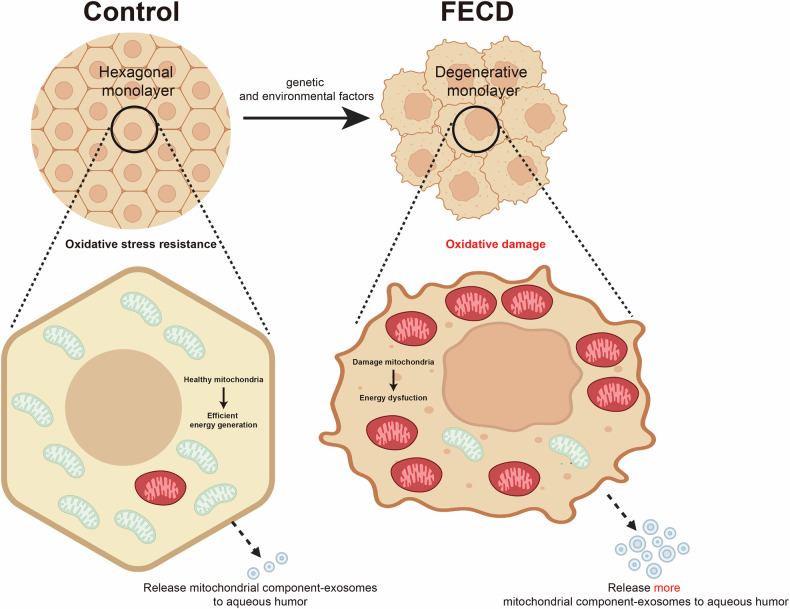
Schematic illustration of corneal endothelial cells releasing mitochondrial components to aqueous humor via extracellular vesicles to deal with excessive oxidative stress caused by Fuchs endothelial corneal dystrophy (The schematic diagram is created with BioRender.com).

In this study, we found that CECs can release components of mitochondria via extracellular vesicles, and it was more pronounced under FECD conditions (Fig. 7). Our data indicates that CECs are able to mitigate mitochondrial stress burden, which is the mechanism of choice for mitochondrial quality control in CECs. To the best of our knowledge, this is the first study to put forward that mitochondria can release from CECs under physiological and pathological conditions. This innovative work would provide theoretical insights and clinical implications for the treatment of FECD.

## Materials and methods

### Patients and ethics

A total of 7 patients with early and middle stage FECD combined with cataract were selected from Shandong Eye Hospital, and 6 age-related cataract patients in the same age group were selected as the control group. AH is extracted from patients before cataract surgery for testing. All patients underwent a detailed and comprehensive examination, excluding other eye and systemic diseases such as glaucoma, conjunctivitis, uveitis, or previous eye surgery, laser, or drug treatment. All informed consent were obtained from all patients. This study followed the principles of the Declaration of Helsinki and was approved by the Ethics Committee of Shandong Eye Hospital with the informed consent of all patients.

### Eye examination

All patients underwent a comprehensive preoperative eye examination, including slit-lamp microscopy (SL-D7; Topcon, Japan), measurements of axial length (AL) and curvature by IOL-Master biometry (Master700, Zeiss, Germany), and measurement of intraocular pressure (IOP) using a handheld rebound tonometer (SW-500, Solvay, China). The corneal endothelium was measured using endothelial microscope (NSP9900II, Konan, Japan) and confocal microscopy (HRT-3, Heidelberg, Germany). This study primarily collected endothelial cells and guttae from the central cornea for analysis. The central corneal thickness was assessed by optical coherence tomography (OCT) (RTVUE XR, Optovue, USA).

### Sample collection and processing

Before cataract surgery, a clear corneal puncture was performed on each patient after surface anesthesia (Alcaine, Alcon, USA), and 100 ul of anterior aqueous humor was extracted with a 29-Gauge insulin syringe (BD Ultra-Fine, China), then transferred to a 1.5 ml cryogenic vial and stored at −80 °C for future use. Any AH sample suspected to be contaminated with blood or iris pigments was discarded.

### Protein extraction and tryptic digestion

The AH from seven FECD patients and six control patients were analyzed using proteomics profiling. The samples were retrieved from −80 °C freezers, and total proteins were subsequently extracted from each. Each sample group was treated with a 1% protease inhibitor for ultrasonic lysis. The supernatant was collected, transferred to a new centrifuge tube, and the protein concentration was determined using the BCA protein assay (BCA Protein Assay Kit, Beyotime, Shanghai, China). The protein from each sample was enzymolized in equal amounts, with the volume adjusted to match the lysate and dithithreitol (DTT) added to achieve a final concentration of 5 mM, and reduced at 56 °C for 30 min. Iodoacetamide (IAA) was then added to achieve a final concentration of 11 mM, followed by incubation at room temperature in the dark for 15 min. The urea was diluted by adding TEAB to ensure a concentration below 2 M. Trypsin was added at a ratio of 1:50 and enzymolized overnight. This was followed by the addition of trypsin at a 1:100 ratio, with enzymolysis continued for 4 h.

### Liquid chromatography-mass spectrometry (LC-MS) analysis

The 4D Label-free quantitative proteomic analysis of AH protein based on LC-MS was performed by PTM-Bio (Hangzhou, China). The peptides were separated using an ultra-high performance liquid phase system, ionized via an NSI ion source, and subsequently analyzed by Orbitrap Exploris™ 480 mass spectrometry (Thermo Fisher Scientific, MA, USA). The scanning range of primary mass spectrometry was set to 400–1200 m/z, and the scanning range of the secondary mass spectrometry was fixed at 110 m/z. Data acquisition was performed using a data-dependent scan (DDA) program, with the dynamic exclusion time for the series mass spectrometry scan set to 30 s to prevent repeated scanning of parent ions. The resultant MS/MS data was processed with the MaxQuant search engine.

### FECD mouse model

Female C57BL/6 mice (7–8 weeks old) produced by Beijing Vital River Laboratory Animal Technology Co., Ltd. were used in the experiment. The nongenetic FECD mouse model was obtained as previously described [29]. Briefly, A UVA light-emitting diode (LED) light source (M365LP1, Thorlabs, USA) was focused to illuminate a 4-mm-diameter spot onto the mouse cornea. The energy was measured using a laser sensor (model L49 150 A, Ophir, Israel), and the time of UVA exposure was adjusted to deliver the appropriate fluency (20 min 57 s for 500 J/cm^2^). The treated eye was irradiated, while the other untreated eye served as control. The mouse experiments were adhered to the guidelines of the Association for Research in Vision and Ophthalmology (ARVO) and approved by the Ethics Committee of Shandong Eye Institute.

### Cell culture and treatment

B4G12 cell line was purchased from Creative Bioarray. The B4G12 line of HCEC was cultured in a medium comprising human endothelial serum-free medium (Creative Bioarray, Grand Island, USA), 2% fetal bovine serum (Gibco, Grand Island, USA), penicillin (100 U/mL), streptomycin (100 mg/L), and human basic fibroblast growth factor (10 ng/mL; R&D Systems, Minneapolis, MN, USA). The cultures were incubated at 37 °C in 5% CO_2_, with the medium being refreshed every two days. All cells used for experiments were cultured for <6 passages. Upon achieving a 60–70% confluence of HCECs, cells were irradiated using two 19.5-inch UVA tubes (XX-15L; Analytik Jena US LLC) emitting light at a wavelength of 365 nm (irradiance: 14.77 mW/cm^2^). The delivered light fluence was 50 J/cm^2^, corresponding to an exposure time of 55 min and 30 s at a distance of 10 cm from the light source. After treatment, the supernatants and HCECs were collected for further applications.

### Extracellular vesicles preparations

EVs were isolated through ultracentrifugation, as described previously [44]. The supernatant was collected and centrifugated at 1000 g for 10 min and clarified to eliminate cell debris and macro-particles. Subsequently, an additional 20 min for 2000 g centrifugation is needed. The supernatants were pooled by ultra-filtration through a 100 kDa molecular weight cut-off membrane (Thermo Fisher Scientific, MA, USA) at 10,000 g for 30 min. The concentrated supernatant was subjected to ultracentrifugation at 110,000 g for 60 min. After re-suspension and ultracentrifugation, the pellet was suspended in PBS for subsequent application. TEM, NTA, and WB were applied to identify the EVs.

### Mitochondrial DNA (mtDNA) copy number analyses

Total DNA was extracted from EVs using the Easy Pure genomic DNA kit (Transgenes, China) according to the manufacturer’s protocol. The copy numbers of mtDNA genes, including MT-COX1, MT-ND1, and MT-ND6, were measured by qRT-PCR using haemoglobin β1 (β-globin) as a reference gene, and presented as a ratio of mtDNA to nuclear DNA. The primer sequences are provided in Supplementary Table S1.

### Mitochondrial tracing virus transfection

To detect the changes in mitochondrial morphology and localization, HCECs were transfected with the Mitochondria GFP (Mito-GFP) tracking lentivirus (Genechem, Shanghai, China) which was diluted to a titer of 1 × 10^8^ TU/ml. Then, 20 μl virus combined with 40 μl HitransG A was added to 1 ml medium in a 6-well plate following the manufacturer’s protocol. The transduction was performed in the culture medium with HCECs for 16 h. The transfected HCECs were exposed to 50 J/cm^2^ UVA, then observed and captured with a laser scanning confocal microscope (LSM 800; Zeiss, Germany) 72 h later after irradiation.

### Enzyme-linked immunosorbent assay

Aqueous humors were collected from FECD patients and controls during cataract surgery, and subsequently homogenized in a PBS solution containing a comprehensive protease inhibitor mixture (Roche, Basel, Switzerland). The expression levels of phosphate carrier protein (SLC25A3) (ZCIBIO, Shanghai, China), pyruvate carboxylase (PC) (Cloud-Clone, Wuhan, China), and parkinson disease protein 7 (PARK7) (Abcam, Cambridge, UK) were quantified utilizing the specified ELISA kits in adherence to the manufacturer’s guidelines.

### Immunofluorescence staining

Corneal samples were obtained from FECD patients undergoing corneal transplantation and donors, which were obtained from our eye banks and were unsuitable for transplantation in humans. The corneas were removed from the corneal medium preservative solution (Optisol, USA), then fixed with 4% paraformaldehyde for 12 min, permeabilized with 0.5% Triton X-100 for 5 min, and subsequently blocked with 2.5% bovine serum albumin (BSA) for 1 h at room temperature. The samples were incubated with TOM20 (1:100, Abcam, ab78547, Cambridge, UK) at 4 °C overnight. Following treatment with secondary antibodies (1:200, Abcam, ab150108, Cambridge, UK) for 1 h at room temperature, the nuclei were stained by 4,6-diamidino-2-phenylindole (DAPI; Beyotime, China). Digital images were visualized and captured utilizing a laser scanning confocal microscope (LSM 800; Zeiss, Germany).

### Transmission electron microscopy analysis

The samples were fixed in 2% glutaraldehyde (Solarbio, Beijing, China) for multiple days, followed by post-fixation using 1% OsO_4_ in 0.1 M cacodylate and 0.5% uranylacetate in 0.05 M maleate buffer. Subsequently, they were dehydrated in varying concentrations of ethanol (ranging from 70% to 99%). The dehydrated samples were infiltrated with a 1:1 mixture of isopropanol and epon overnight, followed by further infiltration into pure epon for an additional 8 h. The samples were cut using an ultramicrotome (Hernalser Hauptstrasse 219, Leica, Austria) equipped with a 45 °C diamond knife (Diatome, AG P.O. Box, Switzerland). The normal thickness of the cut samples is about 40–60 nm, which were placed on a 300-mesh nickel grid and covered with carbon-coated formvar. After staining with uranylacetate and leadcitrate solution, the samples were examined under a transmission electron microscope (CM100, FEI) operating at 80 kV. Image recording and analysis were performed by the Service-bio company (Wuhan, China).

### Live-dead and JC-1 staining

The cell viability staining was performed using a Live-Dead viability staining kit (LIVE/DEAD Viability/Cytotoxicity Kit, Invitrogen, California, USA). HCECs were cultured and obtained after UVA and 20 μM GW4869 (MCE, New Jersey, USA) treatment. Then, the mixed fluorescein-conjugated Etho-1 and the calcein reagent solution were added. In the Live-Dead assay, the dead cells and live cells were examined by fluorescence microscopy. The mitochondrial membrane potential was evaluated using the JC-1 assay kit (Beyotime, Shanghai, China). After the HCECs were treated with UVA and GW4869, 1 ml medium and 1 ml JC-1 dye working solution were added into each well. After incubation at 37 °C for 20 min, the supernatant was discarded, and the cells were washed using JC-1 dye buffer. The cells were observed with fluorescence microscopy.

### Statistical analysis

All experiments were performed at least three biological replicates, with data expressed as mean ± SD. Statistical analysis was performed using GraphPad Prism 8.0. Statistical significance between two groups was determined by Student’s *t*-test. For multiple group comparisons, statistical significance was evaluated using One-way ANOVA followed by Dunnett’s test. *P*-value < 0.05 was considered statistically significant.

## Supplementary information

Primer sequences for RT-PCR. Related to Methods.

Original western blots

## Data Availability

The mass spectrometry proteomics data have been deposited to the ProteomeXchange Consortium via the PRIDE [45] partner repository with the dataset identifier PXD065309. The original Western blots are shown in the “Supplementary Material”.
