# Supplementary material for: Blockade of mitochondrial components release by exosome pathway promotes the pathogenesis of Fuchs endothelial corneal dystrophy
Source: Cell Death Discov. 2025 Dec 2;12:30. doi: 10.1038/s41420-025-02881-3 (PMC12811314; doi:10.1038/s41420-025-02881-3)
Supplement: Supplementary file 1 — Primer sequences for RT-PCR. Related to Methods. [file 41420_2025_2881_MOESM1_ESM.docx]

**Table S1. Primer sequences used in RT-PCR**

| **Genes** | **Sequences** |
| --- | --- |
| MT-ND1 | Forward: 5'-ATGGCCAACCTCCTACTCCT-3' |
|  | Reverse: 5'-GCGGTGATGTAGAGGGTGAT-3' |
| MT-ND6 | Forward: 5'-CCAATAGGATCCTCCCGAAT-3' |
|  | Reverse: 5'-AGGTAGGATTGGTGCTGTGG-3' |
| MT-COX1 | Forward: 5'-GCATATTTCACCTCCGCTACCA-3' |
|  | Reverse: 5'-CCACCTACGGTGAAAAGAAAGATG-3' |
| β-globin | Forward: 5'-CTATGGGACGCTTGATGT-3' |
|  | Reverse: 5'-GCAATCATTCGTCTGTTT-3' |
