# Supplementary figures and images for: Blockade of mitochondrial components release by exosome pathway promotes the pathogenesis of Fuchs endothelial corneal dystrophy
Source: Cell Death Discov. 2025 Dec 2;12:30. doi: 10.1038/s41420-025-02881-3 (PMC12811314; doi:10.1038/s41420-025-02881-3)

**Full and uncropped western blots** **of Figure 5E**


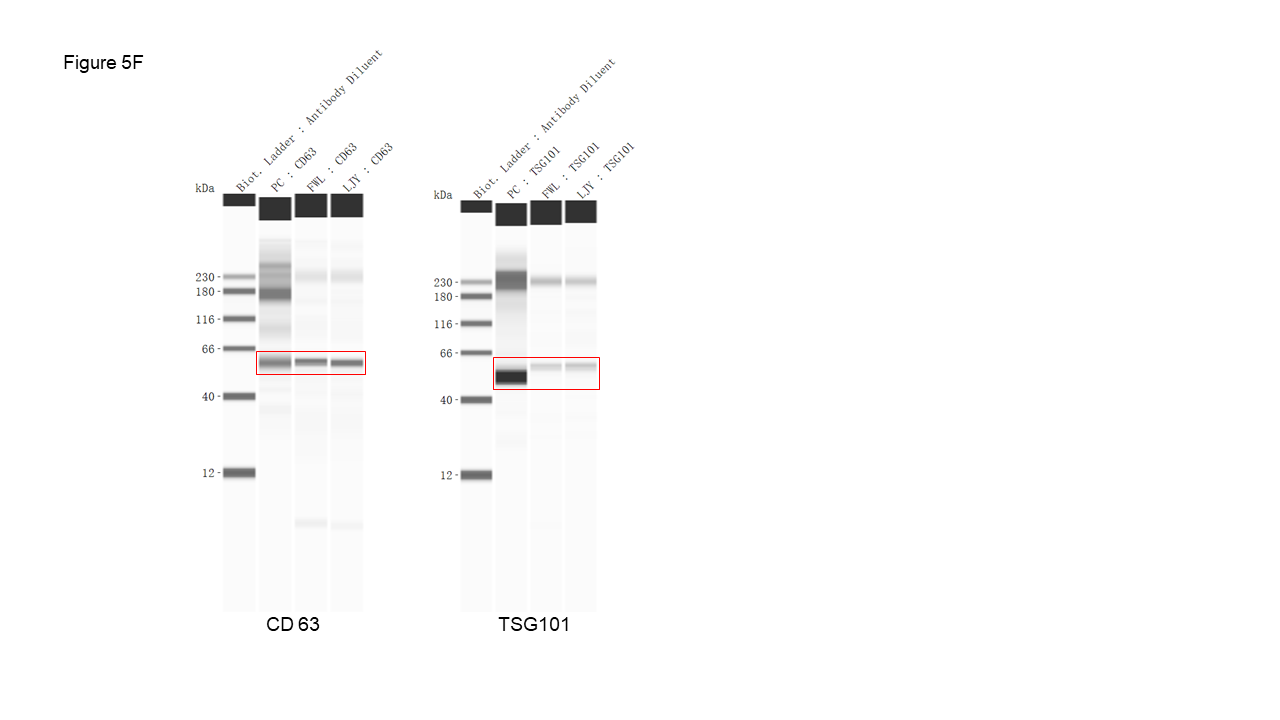

Supplement: Supplementary file 2 — Original western blots [file 41420_2025_2881_MOESM2_ESM.docx]
